# EEG study of the neural representation and classification of semantic categories of animals vs tools in young and elderly participants

**DOI:** 10.1186/1471-2202-14-S1-P318

**Published:** 2013-07-08

**Authors:** Yuqiao Gu, Giulia Cazzolli, Brian Murphy, Gabriele Miceli, Massimo Poesio

**Affiliations:** 1 CLIC, CIMeC - Center for Mind/Brain Sciences, Università degli Studi di Trento, Rovereto (TN), I - 38068, Italy; 2 CeRiN, CIMeC - Center for Mind/Brain Sciences, Università degli Studi di Trento, Rovereto (TN), I - 38068, Italy; 3 School of Computer Science, Carnegie Mellon University, Pittsburgh, PA 15213-3891, USA; 4 School of Computer Science and Electronic Engineering, University of Essex, Colchester, CO7 9QZ, UK

## 

Our semantic knowledge is essential for unimpaired cognition, language and behavior. Previously, we have used Electroencephalography (EEG) to decode the semantic categories of animals vs tools in younger adult subjects during a covert image naming task[1]. In this work, we are extending this approach to older subjects. To date we have recorded EEG from 6 normal older controls. A set of EEG features were extracted from the time-domain at a range of temporal scales. These signal amplitude features were used as inputs to *L2*-penalised logistic regression classifier, after univariate Anova feature selection. Figure 1 compares the classification results of the older group (60-79yrs, mean 68) to the previous cohort of 7 younger participants (25-33yrs, mean 29). The mean accuracy for these two groups increases similarly with the number of channels used as input to the classifier, and reaches a saturation value around 75% (Figure 1A), where baseline is 50%, and an accuracy >56% is significant at α = 0.05. Using a narrow sliding window to extract features (width of 25 ms, moving in steps of 10 ms) over the period 200 ms before to 1000 ms after stimulus onset, we found that the accuracy peaks higher and earlier in the younger subjects (Figure 1B), but the contrast only approached significance with the current sample size. We also calculated the mean classification accuracy of each channel and plotted them as scalp-maps. The results show that the accuracy is higher in right frontal and occipital regions in younger subjects (Figure 1C), while the accuracy is higher in occipital, parietal and left frontal areas in older subjects (Figure 1D).

**Figure 1:**
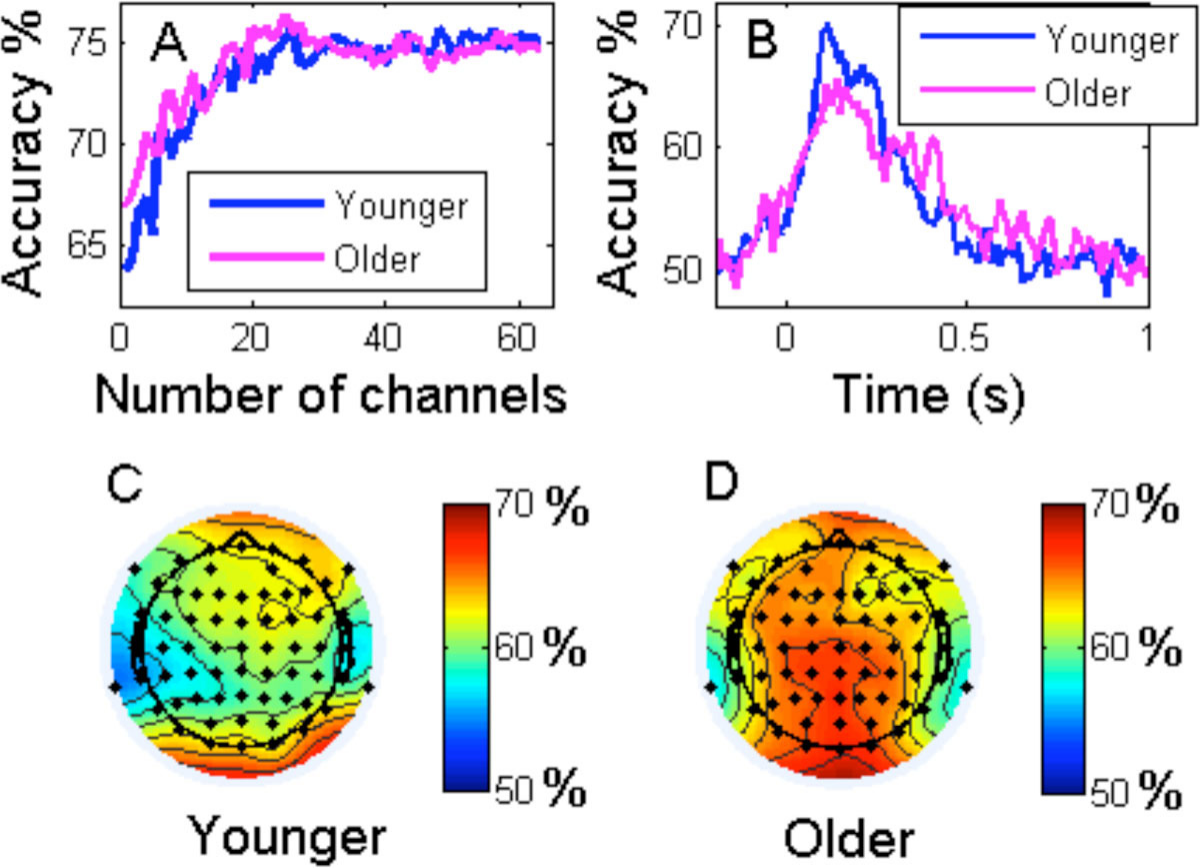
**Comparison of classification accuracy of animals vs tools between younger and older groups**.

## Conclusion

The overall mean classification accuracy of younger and older groups increases similarly with number of channels. Further results indicate that the accuracy of the two groups varies differentially with time and spatial location. We hypothesize that differences in accuracy over time might reflect changes in neuronal and synaptic activity, while differences in accuracy with scalp location indicate that the semantic network might undergo a re-organization process with age increase. To test this hypothesis further recording and analysis are required. Data on elderly controls will serve as reference for clinical applications of EEG recordings in the diagnosis and treatment of language (especially semantic) disorders in neurodegenerative and other diseases of the central nervous system.
